# Unsociability and social adjustment of Chinese preschool migrant children: The moderating role of resilience

**DOI:** 10.3389/fpsyt.2023.1074217

**Published:** 2023-01-24

**Authors:** Jingjing Zhu, Zhenzhen Zhang, Pin Xu, Kaiyu Huang, Yan Li

**Affiliations:** ^1^Early Childhood Education College, Shanghai Normal University, Shanghai, China; ^2^Hongkou District Education College, Shanghai, China; ^3^Qingpu District Education College, Shanghai, China

**Keywords:** preschool migrant children, resilience, unsociability, social adjustment, China

## Abstract

**Objectives:**

The present study examined the moderating effect of children’s resilience on the relations between unsociability and social adjustment (i.e., prosocial behaviors, peer exclusion, interpersonal skills, internalizing problems) in Chinese preschool migrant children.

**Methods:**

Participants were *N* = 148 children (82 boys, *M*_age_ = 62.32 months, *SD* = 6.76) attending two public kindergartens in Shanghai, People’s Republic of China. Mothers provided ratings of children’s unsociability and resilience; teachers assessed children’s social adjustment outcomes, and children reported their receptive vocabulary.

**Results:**

Unsociability was positively associated with peer exclusion and internalizing problems, and negatively associated with prosocial behaviors and interpersonal skills among Chinese preschool migrant children. Moreover, children’s resilience significantly moderated the relationship between unsociability and social adjustment. Specifically, among children with lower levels of resilience, unsociability was significantly and positively associated with peer exclusion and internalizing problems, while among children with higher levels of resilience, unsociability was not associated with social adjustment difficulties.

**Conclusion:**

The current findings inform us of the importance of improving children’s resilience to buffer the negative adjustment among Chinese migrant unsociable young children. The findings also highlight the importance of considering the meaning and implication of unsociability for preschool migrant children in Chinese culture.

## Introduction

In early childhood, peer relationship plays an essential and unique role to children’s social, emotional, and cognitive development (1). Children who do not engage in peer interactions (e.g., socially withdrawn children) may miss out on the potential benefits of interactive experiences, peer support, and other benefits that come from such social situations, which cause a host of behavior problems and adjustment difficulties, exposing those children to additional psychological developmental risks and dilemmas (2). Social withdrawal is defined as the behavior of children who inhibit themselves from participating in peer interactions and exhibit solitary pastimes in social contexts (2, 3). According to the conceptual model proposed by Asendorpf, children’s social withdrawal behavior is determined by a combination of social approach motivations (i.e., desire to seek social interactions) and social avoidance motivations (i.e., desire to avoid social interactions), which can be further classified as shyness, unsociability, and social avoidance (4, 5). Unsociability and shyness are based on different levels of internal approach and avoidance motivations in challenging social situations (6). Shyness is characterized by approach-avoidance motivation conflict, in which shy children want to play with their peers but are simultaneously inhibited by fear and anxiety (5). In contrast, unsociability is characterized as reflecting a combination of low social approach motivation and low social avoidance motivation, referring to behaviors in which children are not interested in social activities and do not actively resist the interaction of others (5). However, shyness and unsociability are positively associated with withdrawal behaviors, as well as negatively associated with social initiations and psychological engagement during peer interactions (7). Shyness and unsociability are characterized as related but distinct constructs and, therefore, it has become common practice to control for any shared variance in order to explore their unique effects and implications (7).

Unlike shyness, unsociability has been considered a relatively benign form of social withdrawal, particularly in early childhood, this might be due to the endorsement of preferred solitary behavior in individualistic societies (7, 8). In support of this notion, results from a study among young children suggested that unsociability is not necessarily associated with perceived social and physiological adjustment (i.e., peer problems, internalizing problems, social anxiety) (7). Because collectivistic cultures society such as China places a high emphasis on social interdependence and group affiliation, parents teach children to form a sense of family affiliation and responsibility from an early age (9). In this regard, unsociable children who pursue their preferences rather than stay in groups may be considered anti-collective and selfish, which may cause adverse reactions from others and increase their risk of negative social adjustment (10). For example, results from previous studies showed that unsociability in Chinese adolescents and preschool children was uniquely associated with peer problems (i.e., peer victimization, peer exclusion), internalizing problems (i.e., loneliness, depression), and academic problems (11, 12).

In recent years, with the accelerated development of the economy and urbanization process in China, rural-to-urban migration has gradually become one of the most salient contextual factors shaping Chinese family life in the 21st century (13). According to the “Annual Report on China’s Education for Migrant Children (2019–2020),” the number of migrant children under 17 years old in China was 34.26 million, among which the size of preschool migrant children under 5 years old had reached 10.53 million, which is accounting for 30.74% of the total number of migrant children, ranking first among all age groups and the most significant increase (14). Furthermore, compared to school-age migrant children, preschool migrant children face enormous challenges, such as the upbringing setting transition from family to kindergarten, the rapid development period of physics and physiology, as well as the adaptation of increasingly complex external environment, which all have caused certain impacts and challenges to cognitive development and interpersonal interactions (15). Thus, we cannot ignore the social adjustment of preschool migrant children.

To our knowledge, the relations between Chinese children’s migrant experience and psychological well-being have been extensive study in the past decade. For example, previous studies indicated that compared with non-migrant children, migrant children are more likely to experience higher prevalence of mental health problems, including depression, social anxiety, and behavior problems (16, 17). Both residential and school mobility represent significant ecological transitions for children, which is a major challenge for the positive adjustment of migrant children. The migrant children’s social adjustment is also influenced by the *hukou* system, which allocates residency rights to the birth population, linking their rights and benefits to their *hukou* status and location. Previous evidence revealed that the preservation of the current *hukou* system of household registration might expose urban migrants to unfair maltreatment and make migrants a vulnerable social group (18). Because migrant parents and children do not have legal registration status in the city. As a result, migrant children do not have the same privileges as urban children, which do not have access to the same quality of education in public schools. In such cases, migrant children may not be able to access stable support from the city, which perhaps leads to their social maladjustment. In addition, it has been found that the screen rate for social withdrawal is higher in migrant children than in non-migrant children in China (19). Thus, migrant children may be more likely to exhibit unsociable behavior. Accordingly, in the present study, we sought to examine the relations of unsociability with social adjustment in Chinese migrant children. To our knowledge, the only existing study related to this topic is the one conducted by Ding et al. (20) on relations between unsociability and adjustment in migrant children in China. The researchers found in that study that unsociability is more evidently associated with social adjustment problems in Chinese migrant children than in non-migrant children.

Notwithstanding, not all unsociable children undergo social maladjustment, implying that some potential risk or protective factors may affect adjustment outcomes for withdrawn children in China (21, 22). The previous study has found that some migrant children follow a “disadvantaged—resilient—well-developed” trajectory, resulting in positive physiological adjustment (23). Resilience, as “the process of, capacity for, or outcome of successful adjustment despite challenging or threatening circumstances,” is regarded as an important protective factor for emotional and behavior problems of children (24). Accordingly, we would focus on unsociability in the current study, which has drawn noticeably less consideration than shyness among Chinese young children (12). More precisely, we explored the potential moderating role of resilience in relations of unsociability with social adjustment in a sample of preschool migrant children in China.

### Unsociability and social adjustment

Cultural contexts play an important role in the development of children’s social behavior and adjustment functioning (9). The attitudes of peers, parents and teachers to specific social behaviors vary across cultures, which influence children’s social, emotional, and school adjustments (25). In western societies, unsociability does not imply low social skills and is sometimes viewed as an expression of personal choice, autonomy, or self-oriented action (8). Thus, unsociability is considered to be a relatively benign form of social withdrawal. In this regard, previous evidence consistently revealed that unsociability is not associated with internalizing problems, peer problems, and social anxiety in children from early childhood through early adolescence (7, 26), and peers also report greater acceptance of unsociable children than shy peers (27). However, the Chinese Confucian culture emphasizes children’s obedience to the expectations and standards of authoritative parents, but unsociable behavior is notably characterized by solitary action, and unsociable children do not inhibit their willingness to continue to indulge in solitary play because of the needs of the surrounding environment, which is clearly at odds with the demands of a collectivist culture (28). Thus, unsociability is more likely to cause negative outcomes on children’s social, psychological, and school adjustment in Chinese society. For example, Liu et al. found that unsociability was associated with adjustment difficulties more strongly in Chinese children than in their Canadian counterparts (29). Furthermore, in a sample of Chinese early adolescents, research evidence suggested that unsociability was associated with peer difficulties, school maladjustment, and internalizing problems (11, 30), even in early childhood (12, 31).

Given that unsociable children experience increased adjustment difficulties, not to mention preschool migrant children in China, it is crucial to identify factors that “protect” or “buffer” against negative outcomes. One area of interest involves the role of resilience, which has been found to alleviate children’s internalizing problems, peer problems, and prosocial behaviors (32). Additionally, previous evidence also indicated that higher levels of resilience help reduce social withdrawal behaviors among Chinese left-behind preschool children (33), but the effect on unsociability remains unclear. While previous study has identified the protective influence of resilience on social adjustment among Chinese children, little research examined the resilience in moderating the relations between unsociability and social adjustment, particularly among rural-to-urban migrant preschoolers in China. Therefore, this study would examine the relations between unsociability and social maladjustment and further explore the moderating role of resilience in these relations among Chinese migrant preschoolers.

### Resilience: A moderator

Resilience is defined as an individual’s flexibility to cope with different difficulties and challenges in life and the ability to recover from adversities and misfortunes (34). Miller found that among students with learning disabilities, compared to non-resilience, those with higher levels of resilience could gain successful experiences, begin to identify areas of strength, and engage in activities with peers (35). This further suggested that when adverse situations or risk factors hinder children’s development, children’s resilience could serve as a protective factor, stimulating their inherent qualities, reducing the harmful effects of adverse situations on children, and making them more adaptable (36). Moreover, resilience has been considered an essential element in positive psychology, and it has been found to be associated with children’s subjective well-being and positive cognitions during childhood (37, 38). For example, children with higher levels of resilience perceive less distress and stress, and are less likely to suffer from anxiety and depression (39). In conclusion, resilience can contribute to positive developmental outcomes for disadvantaged children.

As discussed above, because of the increased risks faced by migrant children, there is a need to identify protective factors that can help them successfully adapt to new environments, and manage the accompanying psychological and adjustment challenges that acculturation encompasses. According to the *protective factor model*, when certain positive internal characteristics are present, individuals will have an immunity to stress, which reduces the negative impact of stress on the individual’s adjustment functioning (40). Thus, resilience could act as a positive inner characteristic that reduces or counteracts the negative impact of risk factors on individual development outcomes. Previous studies found that the protective effects of resilience for disadvantaged children (41–43). For example, Martinez-Torteya et al. (41) have found that in early childhood, resilience played a protective role in maintaining positive adaptive and easy temperamental characteristics in children exposed to domestic violence, compared to their non-resilient counterparts (41). Furthermore, previous research suggested that resilient children enable positive adaptation despite maltreatment (44). Sattler and Gershoff also found that children in poverty who reached higher levels of resilience at entry to kindergarten exhibit similar academic achievement throughout elementary school as children not in poverty (45). Similarly, the findings were found in a study sample of Chinese children. For example, Fan and Fan found that higher levels of resilience could reduce the psychological adjustment difficulties (e.g., depression, loneliness, self-esteem) among Chinese left-behind children (42). They identified resilience as a quality necessary for the growth of children in adversity. It has been found that resilience moderated the relationship between peer victimization and depression among migrant school-age children in China (43). Specifically, the negative impact of peer victimization on depressive symptoms decreased with the increased levels of resilience.

In conclusion, most of the existing literature generally points to the positive development of disadvantaged children from a resilience perspective, and little work has yet examined the moderating role of resilience in the relationship between unsociability and social adjustment among unsociable migrant preschoolers in the context of Chinese culture.

### The present study

As mentioned above, unsociability is incompatible with the traditional Chinese values of social interdependence and group affiliation (10). As such, unsociability has been found to cause a range of children’s adjustment difficulties (12, 31), and it could be inferred that migrant unsociable children would face greater adjustment difficulties. According to the *stress-buffer model* (46, 47), although migrant unsociable children experience double distress of life environment and psychological problems, considering resilience as the potential quality for children could buffer the effects of “migration” and “unsociability,” then they would show the positive social adjustment. Indeed, existing studies have indicated that resilience could reduce migrant children’s adjustment problems (23, 32, 48). Previous studies have focused on the impact of relative unsociability and social adjustment difficulties among Chinese non-migrant children (12, 31). However, the underlying mechanism of resilience in the relations between unsociability and social adjustment is unclear among Chinese preschool migrant children (20). Furthermore, previous research has found that compared to unsociable girls, unsociable boys would be less accepted by their peers, experience more peer problems, and have poorer quality of friendships (49). Thus, we examined the gender differences in the subsequent analysis. In addition, children’s social communicative competence plays a vital role in children’s peer experiences (50). It has been indicated that children’s receptive vocabulary is associated with socioemotional adjustment (51). Therefore, it is critical to control for migrant children’s receptive vocabulary in the study.

**FIGURE 1:**
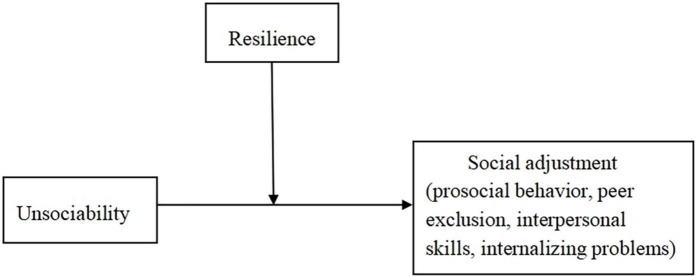
The hypotheses model.

In summary, the primary purpose of the present study was to examine the moderating role of resilience between unsociability and social adjustment among preschool migrant children in China. To be consistent with the previous research (e.g., 11, 12), we focused on four main aspects of social adjustment: prosocial behaviors, peer exclusion, interpersonal skills, and internalizing problems. Based on the extant literature, we expected that unsociability was significantly associated with adjustment difficulties. Furthermore, it was hypothesized that resilience would moderate the relations between unsociability and social adjustment. While at higher levels of resilience, it would serve to buffer unsociable migrant preschoolers from experiencing social maladjustment (see Figure 1).

## Materials and methods

### Participants

Participants were *N* = 148 preschool migrant children (82 boys, *M*_age_ = 62.32 months, *SD* = 6.76) recruited from two public kindergartens in Shanghai, People’s Republic of China. All children were of Han ethnicity, which represents over 97% of China’s population.

Nearly 22% of the mothers and 24% of the fathers had completed high school; 40% of the mothers and 27% of the fathers had completed junior college; 35% of the mothers and 41% of the fathers had earned a bachelor’s degree, and 3% of the mothers and 8% of the fathers had earned a postgraduate degree. Maternal and paternal scores were averaged to create a broader measure of parental education (with higher scores representing higher education).

### Procedure

The present study was reviewed and approved by the ethics review board of Shanghai Normal University. Written consent was obtained from parents of all migrant preschoolers. The participation rate was 98%. Mothers rated their children’s unsociability and resilience. During children’s testing sessions, we assessed children’s receptive vocabulary. Teachers completed measures of children’s social adjustment.

### Measures

#### Maternal ratings

Mothers completed the Chinese version of Child Social Preference Scale (CSPS) (31, 52). Of particular interest was the subscale assessing *unsociability*, which comprises 4 items (e.g., “My child is just as happy to play quietly by his/herself than to play with a group of children,” α = 0.65). Given the common conceptual overlap and similar patterns of adjustment among Chinese youth, it is important to control for any common variation with shyness when exploring the implications of unsociability among Chinese migrant children (29, 52). As such, mothers also completed the *shyness* subscale, which comprises 7 items (e.g., “My child seems to want to play with other children, but is sometimes nervous to,” α = 0.86). Items were rated on a five-point scale (from 1 = “not at all” to 5 = “a lot”). These items were aggregated to create the unsociability and shyness score, with higher scores indicating higher levels of unsociability and shyness.

Mothers also completed the Chinese version of Ego-Resiliency Scale (ERS) (53). The ERS scale comprises 11 items (e.g., “Freezes up when things are stressful, or else keeps doing the same thing over and over again”; α = 0.89). Items were rated on a nine-point scale (from 1 = “not at all” to nine = “a lot”), with higher scores indicating higher levels of resilience.

#### Teacher ratings

Teachers were asked to completed the Chinese version of Child Behavior Scale (CBS) (54, 55). Of particular interest were subscales assessing *prosocial behaviors* (6 items, e.g., “Help other children”; α = 0.90) and *peer exclusion* (7 items, e.g., “Not welcomed by other children”; α = 0.86). Items were rated on a three-point scale (from 1 = “doesn’t apply” to 3 = “certainly applies”), with higher scores indicating higher levels of prosocial behaviors and peer exclusion. The CBS has been shown to be reliable and valid in young Chinese children (55).

Teachers also completed the Chinese version of Social Skills Teacher Rating System (SSTRS) (56, 57). We were particularly interested in the subscales assessing *interpersonal skills* (11 items, e.g., “Make friends easily”; α = 0.90) and *internalizing problems* (4 items, e.g., “Looks lonely”; α = 0.66). Items were rated on a three-point scale (from 0 = “never” to 2 = “always”), with higher scores indicating higher levels of interpersonal skills and internalizing problems. The SSTRS has been shown to be reliable and valid in young Chinese children (57).

#### Children assessments

Children’ *receptive vocabulary* was assessed using the Chinese version of the Peabody Pictures Vocabulary Test (PPVT-III) (58). The scale consists of 204-items, for every item include four pictures, a picture was shown on a quadrant, and the child was asked to identify the picture that best fit the word that was read to the child. Testing took place in blocks of eight items. If a child made more than six errors within a single block of eight items, testing was discontinued. The final receptive-vocabulary scores (range from 0 to 204) were computed by subtracting all the incorrect and missed answers from the total number of items (i.e., 204), and with higher scores indicating higher levels of receptive-language skills (59). The PPVT-III has been shown to be reliable and valid in Chinese children (12).

### Statistical analysis

We used SPSS 26.0 software for data analysis. Preliminary analyses included a series of *t*-tests to explore gender differences and correlations among study variables. Next, we used the PROCESS macro (Model 1) with non-parametric bootstrapping with 1,000 resamples to explore moderating effect (60). The significant effects were probed with a 95% bias-corrected confidence interval (CI) (61). Finally, the Johnson-Neyman (J-N) technique was used to probe significant interactions (62), as suggested by other researchers (49).

## Results

### Preliminary analyses

Descriptive statistics and correlations for all study variables are displayed in Table 1. The results of the *t*-tests indicated that there were significant gender differences in prosocial behaviors (*M*_*boy*_ = 2.24, *SD* = 0.57; *M*_*girl*_ = 2.45, *SD* = 0.52, *t* = −2.27, *p* = 0.02), peer exclusion (*M*_*boy*_ = 1.23, *SD* = 0.43; *M*_*girl*_ = 1.07, *SD* = 0.20, *t* = 2.72, *p* = 0.007), and interpersonal skills (*M*_*boy*_ = 1.36, *SD* = 0.44; *M*_*girl*_ = 1.57, *SD* = 0.36, *t* = −3.11, *p* = 0.002). There were no gender differences in internalizing problems (*M*_*boy*_ = 0.23, *SD* = 0.33; *M*_*girl*_ = 0.14, *SD* = 0.25, *t* = 1.66, *p* = 0.10), resilience (*M*_*boy*_ = 6.36, *SD* = 1.02; *M*_*girl*_ = 6.62, *SD* = 1.02, *t* = −1.55, *p* = 0.12), and unsociability (*M*_*boy*_ = 1.76, *SD* = 0.57; *M*_*girl*_ = 1.67, *SD* = 0.58, *t* = 0.93, *p* = 0.35).

**TABLE 1:** Descriptive statistics and inter-correlations for all study variables (*N* = 148).

	1	2	3	4	5	6	7	8	9	10	11
1. Gender	-										
2. Age (month)	-0.06	-									
3. Parental education	0.02	0.04	-								
4. Receptive vocabulary	0.10	0.52***	0.11	-							
5. Shyness	0.01	-0.07	0.02	-0.19*	-						
6. Unsociability	-0.08	-0.10	0.07	-0.25**	0.63***	-					
7. Prosocial behavior	0.19*	0.46***	0.10	0.53***	-0.27**	-0.29***	-				
8. Peer exclusion	-0.22**	0.01	0.04	-0.22**	0.20*	0.25**	-0.51***	-			
9. Interpersonal skills	0.25**	0.19*	0.08	0.50**	-0.30***	-0.32***	0.63**	-0.65***	-		
10. Internalizing problems	-0.14	0.10	0.09	-0.19*	0.24**	0.15^+^	-0.25***	0.35***	-0.42***	-	
11. Resilience	0.13	-0.11	0.36***	0.14	-0.20*	-0.19*	0.03	-0.11	0.16	-0.07	-
M	-	62.32	-	73.02	1.84	1.72	2.34	1.16	1.45	0.19	6.48
SD	-	6.76	-	28.98	0.68	0.57	0.56	0.36	0.42	0.30	1.02

^+^*p* < 0.01, *p < 0.05, **p < 0.01, ***p < 0.001.

As indicated in Table 1, Unsociability was significantly and positively associated with peer exclusion and internalizing problems (marginal significant), and was significantly and negatively associated with prosocial behaviors and interpersonal skills. Resilience was not significantly associated with indices of social adjustment. Children’s age was significantly and positively associated with prosocial behaviors and interpersonal skills. Children’s receptive vocabulary was significantly and positively associated with prosocial behaviors and interpersonal skills, and significantly and negatively associated with unsociability, peer exclusion, and internalizing problems. Parental education was significantly and positively associated with resilience. Accordingly, we controlled for child gender, age, receptive vocabulary, parental education, shyness in the subsequently analyses.

### Unsociability, resilience, and social adjustment

The primary goal of current study was to examine the moderating effects of resilience in the relations between unsociability and social adjustment among preschool migrant children. To accomplish this goal, we tested the effects of unsociability and resilience (and their interaction) in relation to the outcome variables (i.e., prosocial behaviors, peer exclusion, interpersonal skills, internalizing problems), while controlling for children’s gender,^1^ age, receptive vocabulary, shyness, and parental education. Analyses were conducted using the SPSS macro PROCESS (60). Results are displayed in Table 2. These findings were largely consistent with the correlational analyses (despite the additional control variables). Of particular interest, there were significant interaction effects between unsociability and resilience were also found to be related to peer exclusion and internalizing problems.

**TABLE 2:** Main and moderating effects of unsociability and resilience on indices of social adjustment.

	Social adjustment variables					
	**B**	**SE**	***t* value**	**95% CI**	***R* ^2^**	***F***
**Prosocial behavior**						
Gender	0.33	0.13	2.46*	[0.06, 0.60]		
Child age (month)	0.04	0.01	3.64***	[0.02, 0.07]		
Parental education	0.13	0.09	1.34	[–0.06, 0.31]		
Receptive vocabulary	0.01	0.003	3.52***	[0.004, 0.02]		
Shyness	-0.21	0.12	-1.66	[–0.45, 0.04]		
Unsociability	-0.11	0.09	-1.31	[–0.28, 0.06]		
Resilience	-0.09	0.07	-1.14	[–0.23, 0.06]		
Unsociability × Resilience	0.07	0.06	1.09	[–0.05, 0.20]	0.41	11.90***
**Peer exclusion**						
Gender	-0.33	0.16	-2.05*	[–0.64, -0.01]		
Child age (month)	0.01	0.01	0.85	[–0.02, 0.04]		
Parental education	0.09	0.11	0.84	[–0.12, 0.31]		
Receptive vocabulary	-0.01	0.003	-1.77	[–0.01, 0.001]		
Shyness	0.10	0.15	0.67	[–0.19, 0.39]		
Unsociability	0.14	0.10	1.36	[–0.06, 0.34]		
Resilience	-0.04	0.09	-0.43	[–0.21, 0.14]		
Unsociability × Resilience	-0.17	0.07	-2.26*	[–0.31, -0.02]	0.17	3.61***
**Interpersonal skills**						
Gender	0.37	0.14	20.64**	[0.09, 0.65]		
Child age (month)	-0.01	0.01	-0.45	[–0.03, 0.02]		
Parental education	-0.05	0.10	0.49	[–0.15, 0.24]		
Receptive vocabulary	0.01	0.003	4.92***	[0.01, 0.02]		
Shyness	-0.23	0.13	-1.77	[–0.48, 0.03]		
Unsociability	-0.10	0.09	-0.11	[–0.28, 0.08]		
Resilience	0.004	0.08	0.05	[–0.15, 0.16]		
Unsociability × Resilience	0.09	0.06	1.40	[–0.04, 0.22]	0.35	9.52***
**Internalizing problems**						
Gender	-0.17	0.16	-1.06	[–0.48, 0.15]		
Child age (month)	0.03	0.01	2.21*	[0.003, 0.06]		
Parental education	0.15	0.11	1.33	[–0.07, 0.36]		
Receptive vocabulary	-0.01	0.003	-2.47*	[–0.01, -0.002]		
Shyness	0.31	0.15	2.15*	[0.03, 0.60]		
Unsociability	-0.04	0.10	-0.38	[–0.24, 0.16]		
Resilience	-0.001	0.09	-0.002	[–0.17, 0.17]		
Unsociability × Resilience	-0.18	0.07	-2.94**	[–0.32, -0.04]	0.18	3.93***

*p < 0.05, **p < 0.01, ***p < 0.001.

**FIGURE 2:**
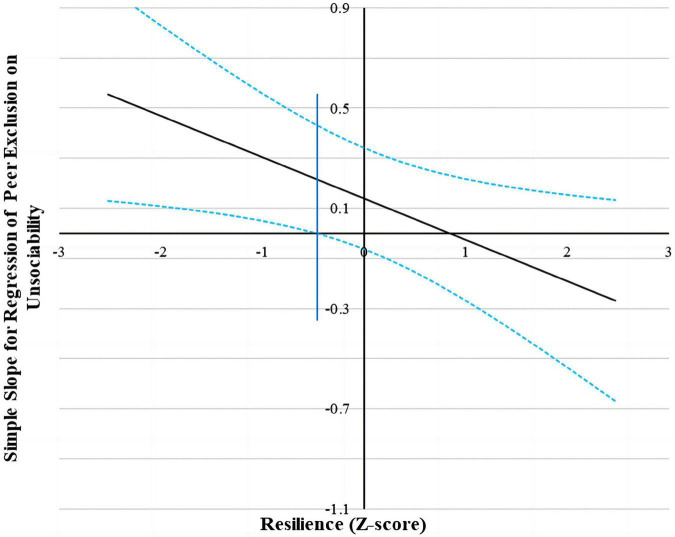
Johnson–Neyman regions of significance and confidence bands for mother-rated unsociability along resilience in relation to peer exclusion. Solid diagonal line represents the regression coefficient for unsociability along resilience. Dashed diagonal blue lines are confidence bands–upper and lower bounds of 95% confidence interval for unsociability regression coefficient along resilience. The vertical blue line indicates the point along resilience at which the unsociability regression coefficient transitions from statistical significance (left of dashed vertical line) to non-significance (right of dashed vertical line). The value of the dashed vertical line is −0.46.

**FIGURE 3:**
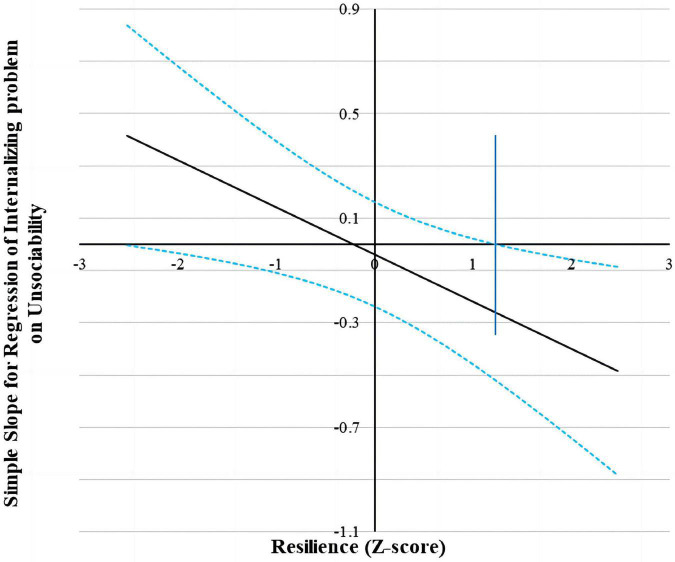
Johnson–Neyman regions of significance and confidence bands for mother-rated unsociability along resilience in relation to internalizing problems. Solid diagonal line represents the regression coefficient for unsociability along resilience. Dashed diagonal blue lines are confidence bands–upper and lower bounds of 95% confidence interval for unsociability regression coefficient along resilience. The vertical blue line indicates the point along resilience at which the unsociability regression coefficient transitions from non-significance (left of dashed vertical line) to statistical significance (right of dashed vertical line). The value of the dashed vertical line is 1.22.

Following suggestions by Hayes and Matthes (63), we used the Johnson–Neyman (J-N) technique to further probe the significant interactions (all the predictors were standardized for the analyses) (62). This technique allowed us to estimate a region of significance for the simple slope of a predictor conditioned on the value of the continuous moderator. The results found that for the prediction of peer exclusion and internalizing problems (see Figures 2, 3), when resilience level was lower than −0.46 SD and 1.22 SD, separately, unsociability was significantly and positively associated with peer exclusion and internalizing problems. However, when resilience level was higher than −0.46 SD and 1.22 SD, separately, unsociability was no longer associated with peer exclusion and internalizing problems.

## Discussion

The goal of the present study was to explore the relations between unsociability and social adjustment and the moderating role of resilience in a sample of preschool migrant children in China. Our results indicated that unsociability was associated with prosocial behaviors, interpersonal skills, peer exclusion, and internalizing problems. Additionally, resilience could be viewed as a protective factor that buffers the negative effects of unsociability on peer exclusion and internalizing problems. To our knowledge, this was the first study revealing the moderate role of resilience in the relations of unsociability with social adjustment, which constitutes significant contribution to our understanding of the mechanism between social behaviors and adjustment in different contexts.

### Association between unsociability and social adjustment

Results from the current study suggested that unsociability was significantly and negatively associated with prosocial behaviors and interpersonal skills, and significantly and positively associated with peer exclusion and internalizing problems (marginal significance) among Chinese preschool migrant children. The findings were similar to the results of Liu et al. (11), who reported that unsociability in Chinese non-migrant children and adolescents was significantly related to peer preference, peer victimization, internalizing problems, learning problems, and loneliness (11). Zhu et al. (12) also reported that unsociability was related to peer exclusion, asocial behaviors, and anxious-fearful among Chinese preschool non-migrant children (12). However, as indicated by recent research, among non-migrant preschoolers in urban settings, unsociability was unrelated to interpersonal skills (64). For migrant children, the life changes and stressful events caused by migrating from rural-to-urban areas may expose children to challenges that affect various aspects of their development, such as their family relationships, peer relationships, and social competence. This study also supported the argument that compared with non-migrant unsociable preschoolers, migrant unsociable preschoolers have poor social skills and may suffer more social adjustment difficulties.

Chinese society appears to be in flux. It has been argued that social change has positively impacted the valuation of unsociability, shifting attitudes closer to Western society’s prevalent view (28). However, Chinese culture emphasizes interdependence and maintaining group harmonization (3). The solitary, non-interacting behaviors have been perceived as undesirable for social regulation and valuation, contrary to the goal of social integration. Therefore, unsociable children are more likely to be negatively evaluated by peers, parents, and teachers, which affects children’s social, emotional, and school adjustment (10). In particular, previous evidence revealed that traditional Chinese values may be maintained to a greater extent in families and children with a rural background in China (20). Accordingly, the conflicts between social change and cultural traditions constitutes a more undesirable environment for unsociable children in social adjustment. Overall, unsociability was associated with social adjustment problems in migrant unsociable children in the present study. The results suggested that migrant unsociable preschoolers in urban settings are more likely to experience adjustment difficulties.

### Moderating effect of resilience

To be consistent with our hypothesis, this study further verified the moderating role of resilience. After controlling for gender, age, receptive language, parental education, and shyness, the results indicated that resilience moderated the association between unsociability and peer exclusion and internalizing problems in Chinese migrant preschool children. Specifically, unsociability had a significant negative predictive effect on peer exclusion and internalizing problems when children reported lower levels of resilience. However, that unsociability had a non-significant predictive effect on peer exclusion and internalizing problems when migrant children reported higher levels of resilience.

Such results support the *protective factor model* of resilience, which suggests that resilience serves as an individual protective characteristic that could buffer the deleterious effects of personal and/or contextual factors (40). In this regard, resilience, which refers to the capacity of individuals to sustain competent functioning or cope successfully with significant change, adversity, or risk may play a vital role in helping unsociable migrant preschoolers to reduce their risk of social maladjustment (34, 43). Accordingly, Michele et al. also found that children with higher levels of resilience displayed more positive emotions, which plays an important role in the individual’s ability to cope with stress (65). Previous evidence revealed that socially avoidant preschoolers with higher levels of emotion regulation would experience fewer internalizing problems (66). Zhao et al. also found that higher levels of resilience mitigated the negative effect of less social support on migrant children’s depression and loneliness (23). Thus, Chinese migrant preschoolers with higher levels of resilience may exhibit fewer internalizing problems. Due to their status as “newcomers” in the urban setting (e.g., school, neighborhood), migrant preschoolers are often discriminated against because they are regarded to have undesirable characteristics (e.g., unfamiliar customs, undesirable behaviors) that mark them as different and then lead them to be rejected, victimized, or excluded by peers (67). Of note, children with higher levels of resilience are more curious, enjoy exploring, and exhibit stronger creativity in playing, which can be accepted and appreciated by peers (54). Previous evidence also indicated that higher levels of resilience could buffer the negative effects of peer victimization among rural-to-urban migrant school-age children in China (43). Consequently, Chinese migrant preschoolers with higher levels of resilience may be less excluded by peers.

Moreover, social integration is one of the disadvantages faced by preschool migrant children in the process of “migration,” which makes the screen rate of social withdrawal of migrant children higher than non-migrant children (19). Thus, migrant children may be more affected by unsociable behavior. Previous study suggested that unsociability was associated with school and psychological problems more evidently in migrant children than non-migrant children (20). According to the *stress-buffer model* (46, 47), higher levels of resilience may be a protective factor that buffers against the negative impact of “migration” and “unsociability” on social adjustment among migrant preschool children. The previous evidence also revealed that resilience could be a critical “shield” for migrant children, reducing the adverse effect of peer discrimination and improving their social adjustment (48). Accordingly, findings from the present study suggested we should highlight the positive effect of resilience on social adjustment among unsociable migrant preschoolers in China.

Unexpected but interesting, results were that resilience non-significantly moderated the relations of unsociability with prosocial behaviors and interpersonal skills in Chinese preschool migrant children. Unsociability denotes non-fearful preference for solitude and less interest in initiating peer interactions in childhood (5). Previous studies revealed that preschoolers are also more self-focused and ego-centric, and may not pay as much attention to peers (68, 69). In this regard, unsociable children inherently exhibit less prosocial behaviors and poor interpersonal skills, and are more likely to be negatively evaluated by parents and teachers. Moreover, migrant preschoolers constantly face unfamiliar and challenging settings (15), and there may be some other factors related to prosocial behaviors and interpersonal skills. For example, for migrant preschoolers, the parent-child interactions were characterized as lower frequency, and less interactions time, resulting in lower levels of parent-child closeness and mothers responding to children’s needs in negative and insensitive ways (70). In this case, migrant children could not learn positive interpersonal skills and exhibit less prosocial behaviors (71). Thus, the moderating role of resilience in the relations between unsociability and prosocial behaviors and interpersonal skills may not be demonstrated in migrant preschoolers.

To summarize, for children with higher levels of resilience, the influence of unsociability on social adjustment was more non-significant. For this result, we can suggest that parents, educators, and others are concerned about migrant children’s unsociable behavior. The resilience of migrant children also needs to be developed when providing appropriate interventions to improve the children’s social adjustment.

### Limitations and future direction

This study makes a novel contribution to the extant literature by providing initial evidence to suggest unsociability associated with a unique pattern of social adjustment difficulties in Chinese preschool migrant children, in which resilience was found to be particularly protective about these relations. Thus, the present study provided valuable information about the role of context in preschool migrant children’s social adjustment, and has implications for prevention and intervention. The findings from this research concerning the relations of unsociability with social adjustment in preschool migrant children suggested that certain vulnerabilities associated with migrant status may indicate appropriate targets for prevention and intervention. For example, parents and professionals should pay particular attention to social adjustment difficulties (e.g., internalizing problems, peer exclusion, poor interpersonal skills, less prosocial behaviors) of migrant unsociable children in various domains (e.g., family, school). Teachers should also help migrant unsociable children to actively engage in social interaction in the kindergarten to reduce their adjustment difficulties. In addition, this study found that resilience could be particularly protective factor in reducing social adjustment problems (i.e., internalizing problems, peer exclusion) of unsociable preschool migrant children. Further research will be critical to the development of specialized prevention and invention programs to improve resilience to reduce internalizing problems and peer exclusion, with practical strategies that are effective for preschool migrant children.

Notwithstanding, some caveats should be considered in interpreting the results, with an eye toward future directions. First, this study only examined preschool migrant children in one of the most developed cities in China (i.e., Shanghai), which is substantial regional differences in social and economic development. Therefore, whether the study results are generalizable remains to be proven. Thus, we can conduct research in other cities with different economic and cultural backgrounds in the future. Second, as the preschoolers can have difficulties reporting their motivations and cognitions (72), mother provided ratings of unsociability as well as assessment of resilience of migrant preschoolers. Mothers’ reports can, to some extent, avoid the subjectivity of children’s self-reports and thus assess children’s social preferences relatively objectively (31). Future research should continue to seek to demonstrate associations between mother-rated unsociability and relevant constructs assessed *via* other means, including direct observation and children’s self-reports. Third, this study was a cross-sectional survey, significantly limiting our ability to make causal inference and to establish the direction of effects. In addition, we only examined the development of unsociability, social adjustment, and resilience in current preschool migrant children, which is far from sufficient. In future, a long-term survey is needed to explore the developmental mechanisms in preschool migrant children. Although more difficult to operationalize, future research is worthwhile to fill this gap.

## Conclusion

This study focuses on the relations between unsociability and social adjustment (i.e., prosocial behaviors, peer exclusion, internalizing problems, interpersonal skills), and the moderating effect of resilience in preschool migrant children in China. The results indicated that unsociability is positively associated with social adjustment difficulties. In addition, consistent with the hypothesis, resilience moderated the relations of unsociability with peer exclusion and internalizing problems among Chinese migrant preschoolers. Specifically, the relations between unsociability and peer exclusion and internalizing problems were more negative among children with lower levels of resilience, but not significant for children with higher levels of resilience. Therefore, this study suggested that unsociable children with higher levels of resilience could mitigate their social adjustment difficulties. The results provided some implications for exploring the occurrence, development, and intervention of unsociability in disadvantaged migrant preschoolers. For migrant unsociable children who are more willing to be alone and seek less outside support, perhaps helping them improve resilience could be considered by parents and educators.

## Data availability statement

The raw data supporting the conclusions of this article will be made available by the authors, without undue reservation.

## Ethics statement

The studies involving human participants were reviewed and approved by Shanghai Normal University. Written informed consent to participate in this study was provided by the participants’ legal guardian/next of kin.

## Author contributions

JZ and ZZ managed the literature search and analyses. PX and KH participated in data collection. YL designed the study. All authors contributed to the article and approved the final manuscript.

## Footnotes

1 There were no significant unsociability × gender interaction effects.
